# PEG-PLGA Co-Loaded Baicalin Mitigates Bovine Viral Diarrhea Virus-Induced Oxidative Stress and Inflammatory Responses Through Modulation of Autophagy and Attenuation of the NLRP3/Pyroptosis Regulatory Axis

**DOI:** 10.3390/biom16040502

**Published:** 2026-03-27

**Authors:** Yanchao Xing, Yingshan Jiang, Ting Ren, Aoyun Li, Yue Teng, Yanlu Li, Junxia Ma, Naichao Diao, Kun Shi, Jianming Li, Ying Zong, Rui Du

**Affiliations:** 1 College of Chinese Medicinal Material, Jilin Agricultural University, Changchun 130118, China; 2 Laboratory of Production and Product Application of Sika Deer of Jilin Province, Jilin Agricultural University, Changchun 130118, China; 3 College of Agriculture, Yanbian University, Yanji 133002, China

**Keywords:** bovine viral diarrhea virus (BVDV), baicalin, PEG-PLGA, NLRP3 inflammasome, anti-virus activity, pyroptosis

## Abstract

Bovine viral diarrhea virus (BVDV), a globally persistent pathogen, causes bovine viral diarrhea-mucosal disease (BVD-MD), a contagious bovine disease posing significant pressures on both public health and economic development. Baicalin (BA), a flavonoid derived from *Scutellaria baicalensis*, exhibits broad antiviral activities but suffers from poor aqueous solubility and low bioavailability, limiting its therapeutic potential against BVDV. To address this limitation, we developed BA-loaded poly (ethylene gly-col)-poly (lactic-co-glycolic acid) (PEG-PLGA) nanoparticles (BA-PEG-PLGA NPs). While autophagy and NLRP3 inflammasome activation have been individually implicated in viral pathogenesis, their functional crosstalk during BVDV infection remains uncharacterized. Herein, we evaluated the antiviral efficacy of BA-PEG-PLGA NPs through integrated in vitro and in vivo experiments. We employed quantitative polymerase chain reaction (qPCR), transcriptome sequencing, Western blot analysis, immunofluorescence microscopy, flow cytometry, and enzyme-linked immunosorbent assay (ELISA) to investigate the mechanisms by which BA and BA-PEG-PLGA NPs combat bovine viral diarrhea virus (BVDV) infection. We found that both free BA and BA-PEG-PLGA NPs effectively attenuated BVDV replication in vitro and in vivo; notably, the nano-formulation exhibited superior efficacy. Mechanistically, BA and its nano-formulation restored autophagy homeostasis, suppressed ROS overproduction, and blocked NLRP3 inflammasome activation and pyroptotic cell death effects comparable to the specific NLRP3 inhibitor MCC950. These findings establish the autophagy–NLRP3/pyroptosis axis as a critical pathogenic mechanism in BVDV infection and reveal that nano-formulated baicalin represents an antiviral strategy by coordinately targeting this axis. This work not only provides a translatable nanomedicine approach for BVDV control but also expands the mechanistic understanding of flavonoid-based interventions in viral inflammatory diseases.

## 1. Introduction

Bovine viral diarrhea virus (BVDV) is a single-stranded positive-sense RNA virus belonging to the genus Pestivirus within the family Flaviviridae. The virus consists of spherical enveloped particles with a diameter of 40–60 nanometers [1]. BVDV is a highly contagious viral disease affecting cattle populations worldwide [2,3]. This virus causes significant economic losses in livestock [4]. The viral replication cycle primarily consists of four stages: adsorption, replication, assembly, and release. The Erns, E1, and E2 proteins play crucial roles in facilitating BVDV entry into host cells [5]. Clinically, BVDV infection leads to inflammatory diarrhea, immunosuppression, enteritis, and mucosal necrosis, and the pathogenic mechanism of BVDV remains inadequately elucidated [6].

Viral infection and replication induce host cellular stress responses, often accompanied by autophagy activation [7]. Autophagy plays a dual role in antiviral immunity: on one hand, the host targets invading viral particles via autophagy pathways to limit their spread [8,9]. On the other hand, certain viruses, including BVDV, have evolved strategies to evade or suppress autophagy, such as interfering with autophagosome formation or inhibiting autophagy-related proteins to promote their own replication [10]. Influenza A virus (IAV)-induced cellular autophagy increases offspring virus production, and IAV NP- and M2-mediated autophagy enhances IAV replication through the regulation of the AKT-mTOR signaling pathway and HSP90AA1 expression [11]. Zika virus (ZIKV) activates autophagy to evade natural host immunity [12]. This active regulation of the host defense mechanism highlights the ‘game’ relationship between the virus and the host in the autophagy pathway.

*Scutellaria baicalensis* Georgi is a traditional Chinese medicinal herb widely used for its heat-clearing and detoxifying properties. Its major bioactive constituent, baicalin (BA), is a flavonoid compound with documented anti-inflammatory, antioxidant, and broad-spectrum antiviral activities [13,14]. Previous studies have demonstrated BA’s inhibitory effects against several viruses, including the dengue virus [15], Newcastle disease virus [16], Marek’s disease virus [17], and the influenza A virus [18]. Scutellarin, a structural analog, was also shown to suppress porcine reproductive and respiratory syndrome virus (PRRSV)-induced proinflammatory cytokine expression and disrupt viral replication [19]. Despite these promising properties, the therapeutic application of BA against viral infections, particularly in the context of BVDV, faces significant pharmaceutical challenges. Its low aqueous solubility (~67 μg/mL), poor membrane permeability (~0.037 × 10^−6^ cm/s), and extensive hydrolysis by intestinal microbiota into less active aglycones substantially limit its systemic bioavailability and antiviral efficacy in vivo [20,21].

To overcome these limitations, nanocarrier-based delivery systems have emerged as a viable strategy to enhance the pharmacokinetic profile of poorly soluble phytochemicals. Among them, poly(ethylene glycol)-poly(lactic-co-glycolic acid) (PEG-PLGA) nanoparticles combine the biodegradability and controlled-release capacity of PLGA with the hydrophilic stealth properties of PEG, thereby improving drug solubility, prolonging circulation time, and facilitating targeted delivery to infected tissues while maintaining favorable biocompatibility [22,23]. Although PEG-PLGA has been applied to deliver various therapeutics, its use for baicalin delivery in the context of pestivirus infection has not been previously reported.

The antiviral mechanism of BA may extend beyond direct viral inhibition to modulation of host cellular responses. Emerging evidence suggests that autophagy and inflammatory signaling are interconnected during viral infection [24]. For instance, Japanese encephalitis virus (JEV) infection triggers autophagy-dependent inflammatory responses, and autophagy inhibition attenuates both viral load and inflammation in vivo [25]. Similarly, in H5N1 avian influenza virus infection, knockdown of autophagy-related genes (e.g., *beclin1*, *atg5*) reduces proinflammatory cytokine production and suppresses NF-κB and p38 MAPK activation [26]. These findings imply a potential crosstalk between autophagy and inflammasome-mediated inflammation in viral pathogenesis. Notably, the NOD-like receptor protein 3 (NLRP3) inflammasome serves as a central regulator of innate immunity, and its aberrant activation can drive pyroptotic cell death and excessive inflammation [25,27]. However, whether BVDV infection dysregulates the autophagy–NLRP3/pyroptosis axis and whether baicalin, particularly in nanoformulated form, can modulate this axis to mitigate viral pathogenesis remains unexplored.

Based on these considerations, we synthesized and characterized baicalin-loaded PEG-PLGA nanoparticles (BA-PEG-PLGA NPs) and evaluated their anti-BVDV efficacy in vitro and in vivo. This study aimed to investigate whether BA and its nanoformulation alleviated BVDV-induced oxidative stress and inflammatory injury through coordinated regulation of autophagy and the NLRP3/pyroptosis pathway. Our findings not only provide mechanistic insights for developing host-directed antiviral strategies against pestiviruses but may also serve as a reference for understanding inflammatory pathogenesis in other members of the Flaviviridae family.

## 2. Materials and Methods

### 2.1. Cell Culture and Virus

Madin–Darby Bovine Kidney cells (MDBK cells) and the BVDV NADL strain with GenBank Accession NC_001461.1 were preserved by the Laboratory of Economic Animal Disease, Jilin Agricultural University, Jilin Province, China. MDBK cells were grown in DMEM (Cytiva, Marlborough, MA, USA) containing 10% fetal bovine serum (Thermo Scientific, Waltham, MA, USA) at 37 °C in a cell incubator containing 5% CO_2_.

### 2.2. Preparation of BA-PEG-PLGA Nanoparticles

Briefly, 5 mg of baicalin was dissolved in 200 μL of PBS (W_1_) (Pufei De Bioceth, Chengdu, China). Subsequently, the solution was introduced into the organic phase, which was prepared by dissolving 25 mg of polyethylene glycol-polylactic acid-polyethylene glycol nanoparticles PEG-PLGA (75:25, MW 18 kDa) in 5 mL of dichloromethane. This mixture was then subjected to sonication using an ultrasonic processor at 100 watts in an ice bath for 3 min, resulting in the formation of a primary emulsion. Next, mix the initial emulsion with 10 mL of 1% sodium cholic acid (W_2_) and stir. The evaporation of the organic solvent promotes the formation of a stable W_1_/O/W_2_ double-encapsulated emulsion. The drug loading efficiency (LE) and encapsulation efficiency (EE) of baicalin in PEG-PLGA were determined using the following calculation methods: LE (%) = (drug quality/total weight) × 100%; EE (%) = (actual encapsulated drug quality weight/total weight of added drug) × 100%.

### 2.3. Characterization of BA-PEG-PLGA NPs

Fourier transform infrared (FTIR) (Bruker Daltonik GmbH, Bremen, Germany) spectroscopy was carried out employing a PerkinElmer Spectrum One FTIR spectrometer. X-Ray Diffraction (XRD) (Shimadzu, Kyoto, Japan) analysis was utilized to identify the form of baicalin encapsulated within the PEG-PLGA nanoparticles. The characterization of baicalin in BA-PEG-PLGA NPs was detected through ultraviolet–visible spectroscopy (UV-vis) (PerkinElmer, Paisley PA4 9RF, UK). The average particle size of BA-PEG-PLGA nanoparticles was evaluated using dynamic light scattering (NanoBrook 90plus Zeta, Brookhaven Instruments Corporation, Holtsville, NY, USA). Additionally, the morphology of the particles was examined via a transmission electron microscope (TEM) (Jeol Ltd., Tokyo, Japan).

### 2.4. Stability and Release of BA-PEG-PLGA NPs

With the aim of determining the cumulative release and stability of BA-PEG-PLGA NPs, they were placed in a constant temperature shaker at 100 r/min at 37 °C and 4 °C, respectively. The BA-PEG-PLGA NPs were sampled at 0, 8, 16, 24, 32, 40, and 48 h in the shaker. After centrifugation at 13,000 r for 30 min, collect the supernatant fluid and determine the cumulative release quantity of BA. Measure the particle size, PDI, and zeta potential (with NanoBrook 90plus Zeta) of BA-PEG-PLGA nanoparticles stored at 4 °C at 0 h and after 360 h.

### 2.5. Virus Titer Assays

Viral titer assessment was performed using the TCID_50_ assay. MDBK cells grown to 80% confluence were seeded into a 96-well plate. Serially diluted virus solutions (10^−1^ to 10^−9^) were inoculated into the cells and incubated at 37 °C for 2 h, with eight replicate wells per dilution. Subsequently, 200 μL of DMEM maintenance medium containing 2% equine serum was added to each well. The plates were incubated at 37 °C in a 5% CO_2_ incubator for 72 h. After microscopic observation of cytopathic effect (CPE), the BVDV TCID_50_ titer was calculated using the Reed–Muench method. The titer values were recorded as lg (TCID_50_) per milliliter [28].

### 2.6. Cell Viability Assay

MDBK cells were seeded in a 96-well plate at a density of 2 × 10^4^ cells per well. The plate was incubated at 37 °C for 24 h to allow cell attachment and growth. After 24 h, predetermined concentrations of BA and BA-PEG-PLGA were added to the wells, followed by incubation under identical conditions for an additional 48 h. Subsequently, 10 µL of CCK-8 reagent was added to each well, and the plate was incubated at 37 °C for 1 h. Following the manufacturer’s protocol, absorbance (OD) was measured at 450 nm using a microplate reader (BioTek Instruments (Agilent Technologies), Winooski, VT, USA). Cell viability was calculated for each group relative to the blank control group. The cell viability was calculated according to the following formula: cell viability (%) = [A (experimental group) − A (blank group)]/[A (control group) − A (blank group)] × 100 [29].

### 2.7. Flow Annexin V-FITC/PI Double Staining Method to Detect Cell Apoptosis

Annexin V-FITC/PI double-labeled flow cytometry was used to detect cell apoptosis. Cells were seeded into 6-well plates at a density of 1 × 10^6^ cells per well. There were 3 replicate wells in each group, and a total of 6 groups were set up. The cells were rinsed with pre-cooled PBS and resuspended in 500 μL of mixed binding buffer. The cells were mixed with 5 μL Annexin V-FITC and 5 μL Propidium Iodide (BB-4101, BestBio Co., Shanghai, China), and they were treated at room temperature for 15 min in the dark. Detection was performed on a flow cytometer (AccuriC6, BD Biosciences, San Jose, CA, USA). A total of 1~5 × 10^5^ cells were collected for each experimental sample [30].

### 2.8. Viricidal Effect and Viral Life Cycle Assay

The stage-specific antiviral activity of BA was evaluated using a time-of-addition assay, a well-established strategy for dissecting compound effects on distinct viral lifecycle stages [31]. In the viral inactivation assay, approximately 1 × 10^5^ infectious units/mL of BVDV suspension was mixed with 25 μg/mL baicalin (BA) or dimethyl sulfoxide (DMSO, control) and incubated at 37 °C for 2 h. The mixture was then washed with phosphate-buffered saline (PBS) and repurified by ultracentrifugation (90,000× *g*) through a 20% sucrose cushion. The purified viral fraction was collected for the determination of TCID_50_. For the virus attachment assay, MDBK cells at 80% confluency were pretreated with 25 mg/mL BA at 37 °C for 2 h, followed by BVDV (MOI = 1) adsorption at 4 °C for 2 h. After washing 3 times with PBS, the cells were then collected, and the lysates were analyzed by RT-qPCR. In the virus internalization assay, BVDV (MOI = 1) was allowed to bind to cells at 4 °C for 2 h. The unbound virus was washed off 3 times with PBS (pH = 3). The cells were treated with BA at 37 °C for 2 h before collection and RT-qPCR quantification. For the viral replication assay, BVDV (MOI = 1) entered cells at 37 °C for 2 h. Non-internalized BVDV particles were removed by washing 3 times with PBS (pH = 3.0). BA treatment for 10 h. The cells were then collected and analyzed by RT-qPCR. Finally, in the virus release assay, after 2 h of BVDV (MOI = 1) infection, the cells were washed three times with PBS and cultured in medium containing 2% fetal bovine serum (FBS) at 37 °C for 10 h. The cells were then treated with BA at 37 °C for 2 h, and progeny viruses in the supernatant were collected. The mRNA levels of the BVDV 5′UTR were analyzed by RT-qPCR [32,33,34].

### 2.9. Enzyme-Linked Immunosorbent Assay (ELISA)

The cells previously acquired were used for the ELISA. An enzyme marker was employed to measure absorbance, aiming to independently quantify the expression levels of inflammatory markers IL-18 and IL-1β, the antioxidant marker MDA, as well as caspase-1 (Beyotime Biotechnology, Shanghai, China) and lactate dehydrogenase (LDH) (A020-2-1, NanJing JianCheng Bioengineering Research Institute, Nanjing, China) [35].

### 2.10. Immunofluorescence Assay

After grouping cells for culture, they were fixed with 4% paraformaldehyde for 15 min and washed three times with PBS. Permeabilization was performed with 0.1% Triton X-100 for 5 min. Cells were blocked with 5% BSA for 1 h to reduce non-specific binding. Primary antibodies against E2 (1:100, VMRD, Rosemont, IL, USA), LC3 (1:250, Proteintech, Rosemont, IL, USA), and GSDMD (1:100, ABclonal, Woburn, MA, USA) were applied and incubated at 4 °C overnight. After PBS washing, FITC-labeled anti-rabbit IgG (1:1000) was added and incubated for 1 h at room temperature. Finally, the cells were incubated with DAPI (Beyotime, Shanghai, China) for 10 min to stain the nucleus, and the fluorescence microscope was used for imaging and observation. According to the protocol of the ROS fluorescent probe kit, we added the culture medium containing DCFH-DA to the cells and incubated them at 37 °C in the dark for 30 min to detect the intracellular ROS level.

### 2.11. Quantitative Reverse Transcription-PCR (qRT-PCR)

We used the Transzol Up Plus RNA Kit (ER501–01) to extract total RNA from cells. Then, we carried out reverse transcription, amplification, and RT-qPCR. Detection of BVDV 5′UTR mRNA Expression in Various Tissues and Blood of Mice Infected with BVDV Using qRT-PCR. The mRNA expression levels of the related genes were calculated by the 2^−ΔΔCT^ method, using the *GAPDH* gene as an internal reference (Table 1).

### 2.12. Western Blotting Analysis

After protein lysis of cells in each group, the samples were centrifuged at 4 °C for 15 min to collect the supernatants. The protein concentration was measured using a BCA kit (Beyotime, Shanghai, China). Subsequently, the proteins were separated by SDS–PAGE and transferred to PVDF membranes. After being blocked with 5% skim milk and washed with TBST, the membranes were incubated with primary antibodies at 4 °C. After washing, the membranes were incubated with secondary antibodies on a horizontal shaker. ECL substrate was applied to the membranes for 30 s in a darkroom. Band intensity was quantitatively assessed using ImageJ software (Version 1.53k) via Western blot.

### 2.13. RNA-Seq Data Analysis

Total RNA was extracted with TRlzol reagent from BDV-infected MDBK cells (Sangon, Shanghai, China), following the manufacturer’s protocol (three biological replicates per group). RNA sequencing was performed for the following groups: control, BA, and BA+BVDV. RNA quantification: Qubit™ RNA HS Assay Kit (Life Technologies, Carlsbad, CA, USA); reverse transcription: HiScript II Reverse Transcriptase Kit (#R223, Vazyme, Nanjing, China). Finally, sequencing was performed using the Illumina Novaseq X Plus platform (Illumina, Inc., San Diego, CA, USA).

### 2.14. Animals

Healthy BALB/c mice (4–8 weeks of age, SPF grade) were bought from Yisi Experimental Animal Technology Corporation (Changchun, China) under license number SCXK (JI) 2023–0002. The Animal Welfare and Ethics Committee (Ethical Review Approval No: 20211011003, 11 October 2021) of Jilin Agricultural University approved all animal experiments. The animals were kept at 25 ± 2 °C, with 12:12 h of light and darkness. BALB/c mice were randomly divided into 6 groups with 10 mice in each group: (1) control group, (2) mTOR inducer RAPA (BVDV + RAPA), (3) BVDV, and (4) BVDV+RAPA+ low-dose 5 mg/kg BA-PEG-PLGA group. (5) BVDV+ RAPA+ medium dose 10 mg/kg BA-PEG-PLGA group. (6) BVDV+RAPA+ high dose 20 mg/kg BA-PEG-PLGA group. Except for the control group, the remaining five groups of mice received intraperitoneal injections of 200 μL viral suspension containing 10^5^ TCID_50_ BVDV. On the second day post-infection, mice in each group received oral administration of different concentrations of baicalin nanoparticle solutions via gavage. The induced group received intraperitoneal injections, while the control group and BVDV-infected group received gavage with an equal volume of physiological saline. After 5 consecutive days of oral administration, all mice were euthanized by cervical dislocation, and blood and tissue samples were collected.

### 2.15. Statistical Analysis

Statistical analysis was performed using GraphPad Prism (V9.5). All quantitative results were expressed as mean ± standard error (SEM), and a Student two-tailed *t*-test was used to evaluate the significance of pairwise comparisons. Three or more values were compared using one- or two-factor ANOVA, as well as a post hoc Tukey analysis; a *p*-value < 0.05 was considered statistically significant.

## 3. Results

### 3.1. Preparation and Characterization of BA-PEG-PLGA Nanoparticles

**Figure 1:**
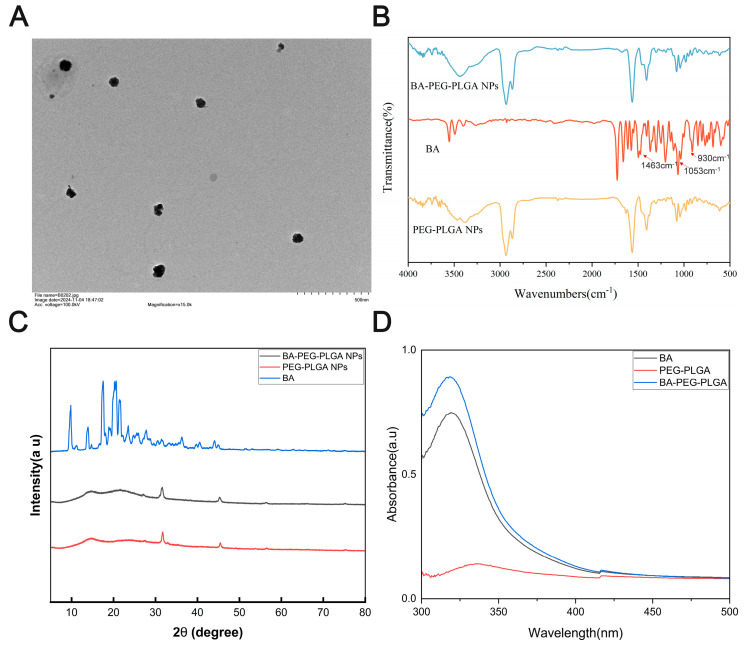

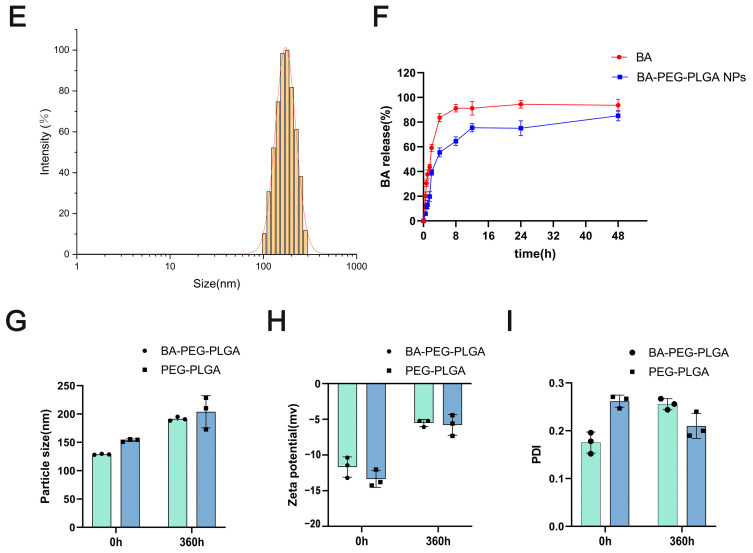
Preparation and characterization of Baicalin-polyethylene glycol-polylactic acid-polyethylene glycol (BA-PEG-PLGA NPs). (**A**) Morphology of BA-PEG-PLGA NPs under the transmission electron microscope. (**B**) Fourier transform infrared (FTIR) spectra of BA-loaded PLGA nanoparticles. (**C**) X-ray diffraction analysis (XRD) patterns of BA, PEG-PLGA NPs, and BA-PEG-PLGA NPs. (**D**) Ultraviolet–visible spectroscopy (UV–Vis) spectra of PEG-PLGA polymer, free BA, and BA-PEG-PLGA NPs. (**E**) Particle size of the BA-PEG-PLGA NPs. (**F**) Cumulative release of BA and BA-PEG-PLGA NPs. (**G**–**I**) are the particle size, zeta potential, and PDI of BA-PEG-PLGA NPs, respectively, Green represents the BA-PEG-PLGA group; blue represents the empty PEG-PLGA nanoparticle group. The data are expressed as the means.

To ensure that the BA-PEG-PLGA NPs possess physicochemical properties suitable for antiviral application against BVDV, we systematically characterized their key attributes that directly influence cellular delivery and therapeutic efficacy. Particle size (<200 nm) and narrow size distribution (low polydispersity index, PDI) were evaluated because they critically determine cellular internalization efficiency and batch-to-batch reproducibility [36]. We measured the zeta potential to evaluate the stability of the nanoparticles during storage and in physiological environments. We quantitatively analyzed the drug’s sustained release kinetics to confirm that the nanoparticles could achieve sufficient drug payload delivery and ensure long-term drug action, thereby inhibiting the viral replication cycle. We verified the successful encapsulation of baicalin through spectroscopic and diffraction analyses. The results of these characterizations are presented in Figure 1, which provides the physicochemical foundation for the subsequent in vitro and in vivo evaluations against bovine viral diarrhea virus (BVDV).

After adding BA to PEG-PLGA, we observed that BA-PEG-PLGA formed smooth spherical particles (Figure 1A). The characteristic absorption peaks of baicalin are located at 1409, 1564, and 1662 cm^−1^, corresponding to the stretching vibration absorption peaks of the benzene ring, while the C-H absorption peaks of aromatic hydrocarbons are distributed between 900 and 650 cm^−1^. In contrast to the characteristic peaks of baicalin in the 500–1700 cm^−1^ fingerprint region, BA-PEG-PLGA shows no significant peaks in this region (Figure 1B). This suggests that the peak shift and reduced peak intensity may be attributed to the encapsulation of baicalin within the PEG-PLGA polymer.

The XRD pattern of baicalin showed distinct peaks between 10 and 40° (2θ), confirming its high crystallinity. The XRD pattern of baicalin exhibits distinct peaks within the 10 to 40° (2θ) range, confirming its high crystallinity. In contrast, baicalin encapsulated within PEG-PLGA nanoparticles shows no crystalline peaks, indicating successful loading of baicalin (Figure 1C). UV-Vis absorption spectra (wavelength 300–500 nm) were scanned. The results (Figure 1D) showed that the BA-PEG-PLGA NPs and BA control all showed absorption peaks at 320 nm, while no obvious absorption peaks were seen for PEG-PLGA at this wavelength, indicating that the UV-visible absorption characteristics of BA were not significantly altered by the encapsulation of BA with PEG-PLGA. The amount of baicalin encapsulated in nanoparticles was found using high-performance liquid chromatography (HPLC). The cumulative release rate of the active pharmaceutical ingredient (API) reached 94.6% within 8 h, achieving nearly complete release. The release rate of baicalin PLGA nanoparticles reached 94.2% within 48 h, indicating that the formulation possesses sustained-release properties (Figure 1F); additionally, the average particle size of BA-PEG-PLGA was approximately 130 nm. The polydispersity index (PDI) of the nanoparticles was 0.175. After 360 h at 4 °C, the particle size did not undergo significant changes, while the negative charge and PDI showed an upward trend (Figure 1E,G–I).

### 3.2. BA-PEG-PLGA NPs Inhibit BVDV Infection in MDBK Cells

**Figure 2:**
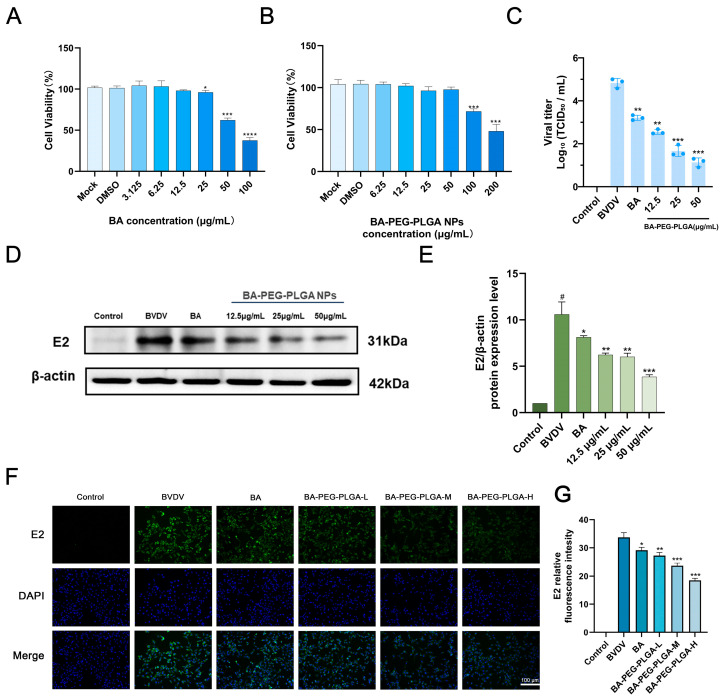
BA-PEG-PLGA NPs downregulated the replication level of BVDV. (**A**) Cytotoxicity of 3.125–100 μg/mL BA in MDBK cells. (**B**) Cytotoxicity of 6.25–200 μg/mL BA-PEG-PLGA NPs in MDBK cells. (**C**) Viral titers were evaluated by the Reed–Muench method. (**D**) The BVDV E2 protein expression levels at post-infection after treatment with 25 μg/mL BA and 12.5, 25, or 50 μg/mL BA-PEG-PLGA NPs. (**E**) The intensity band ratio of intracellular E2 to β-actin. The intensities of the protein bands were quantified using ImageJ. (**F**) Immunofluorescence staining shows BVDV E2 protein (green) and nuclear DAPI counterstain (blue). The scale bar represents 100 μm. (**G**) Relative fluorescence intensity of E2 in MDBK cells after treatment (*n* = 3). Data are presented as means ± SEM of 3 independent experiments. (*n* = 3, # *p* < 0.05 vs. control; * *p* < 0.05, ** *p* < 0.01, *** *p* < 0.001, **** *p* < 0.0001 vs. BVDV). Western blot originals can be found in Figure S1.

CCK-8 assays evaluated cytotoxicity of BA (6.25–25 μg/mL) and BA-PEG-PLGA NPs (12.5–50 μg/mL) in MDBK cells over 48 h, showing no significant viability reduction at tested concentrations (Figure 2A,B). Based on these findings, 25 μg/mL BA, 12.5 μg/mL, and 50 μg/mL BA-PEG-PLGA NPs were selected for subsequent experiments.

The inhibitory effect on viral replication was assessed by measuring the TCID_50_ in the supernatant of MDBK cells infected with BVDV. The BVDV titer was approximately TCID_50_ 10^−4.83^/mL. Following treatment with 12.5 μg/mL BA-PEG-PLGA nanoparticles, the viral titer decreased significantly to approximately TCID_50_ 10^−2.25^/mL (Figure 2C). Notably, high-dose PEG-PLGA-baicalin formulations reduced BVDV titers by approximately 3.7 log_10_ TCID_50_/mL (equivalent to ~5000-fold reduction), indicating potent in vitro antiviral activity. BA, BA-PEG-PLGA-L, BA-PEG-PLGA-M, and BA-PEG-PLGA-H treatments were administered following BVDV infection. This result was further validated by analyzing BVDV E2 protein levels, where 25 μg/mL BA and 50 μg/mL BA-PEG-PLGA nanoparticles significantly inhibited BVDV expression, consistent with TCID_50_ results (Figure 2D,E). Additionally, immunofluorescence analysis (IFA) results confirmed that the inhibitory effect of BA-PEG-PLGA nanoparticles on BVDV infection was dose-dependent, significantly reducing the number of BVDV-infected cells (Figure 2F). These results reflect the activity of BA and BA-PEG-PLGA nanoparticles in inhibiting BVDV replication in vitro. Among these, BA-PEG-PLGA nanoparticles demonstrated superior efficacy to BA at high, medium, and low doses. This is attributed to the nanoparticle formulation’s ability to delay drug release, reduce drug loss, and enhance therapeutic effects.

### 3.3. BA Inhibiting BVDV Replication and Release During the Viral Life Cycle

**Figure 3:**
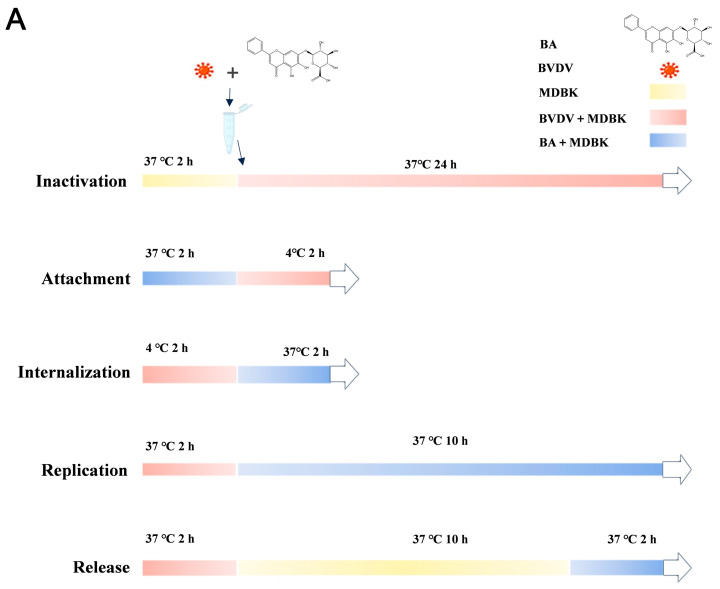

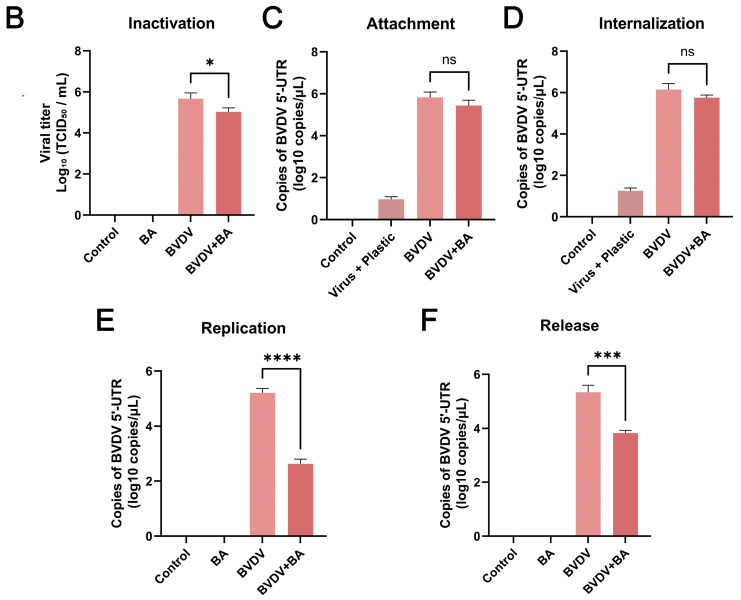
BA inhibits BVDV replication and release. (**A**) Schematic diagram of inactivation, attachment, internalization, replication, and release of BVDV during infection of MDBK cells with 25 μg/mL BA and BVDV. (**B**) Viral inactivation assay: BVDV particles were pre-incubated with BA for 2 h at 37 °C. Following treatment, residual infectious virus was quantified by TCID_50_ assay to determine virucidal activity. (**C**–**F**) Stage-specific antiviral activity of BA assessed by RT-qPCR detection of BVDV 5′UTR mRNA: (**C**) Virus attachment and internalization assay: Control (uninfected MDBK cells), virus + plastic wells (non-specific binding control), BVDV-infected cells, and BVDV+BA cells. All values were background-subtracted using the virus + plastic wells data. Attachment: BA present only during virus adsorption at 4 °C to evaluate blockade of viral binding to host cell receptors; (**D**) internalization: BA added during 37 °C (2 h) to assess interference with clathrin-mediated endocytic entry. (**E**) Replication: BA was added after virus adsorption and removal of unbound virus to determine suppression of intracellular viral RNA synthesis. (**F**) Release: BA was applied at 10 h post-infection (after completion of viral replication) to examine inhibition of progeny virion assembly and egress. (*n* = 3, * *p* < 0.05, *** *p* < 0.001, **** *p* < 0.0001 vs. BVDV; ns, not significant).

In general, the viral life cycle is divided into four steps: attachment, internalization, replication, and release [37]. To clarify the effects of baicalin itself on the replication cycle stages of BVDV. Virucidal activity was determined by the TCID_50_ assay, measuring infectious virus inactivation, and viral inhibition across the BVDV lifecycle was quantified via absolute qRT-PCR of viral RNA copies. The expression of BVDV 5′UTR mRNA was measured by qRT-PCR to further investigate how BA affects the stage of the BVDV life cycle (Figure 3A), which revealed that BA treatment reduced BVDV mRNA levels specifically during replication and release phases compared to untreated virus controls. This indicates BA exerts antiviral effects through interference with viral replication and release stages of the BVDV replication cycle (Figure 3C–F). Notably, BA did not affect viral attachment and internalization (Figure 3C,D) nor directly inactivate viral particles (Figure 3B).

### 3.4. Differential Gene Analysis in Transcriptomics

**Figure 4:**
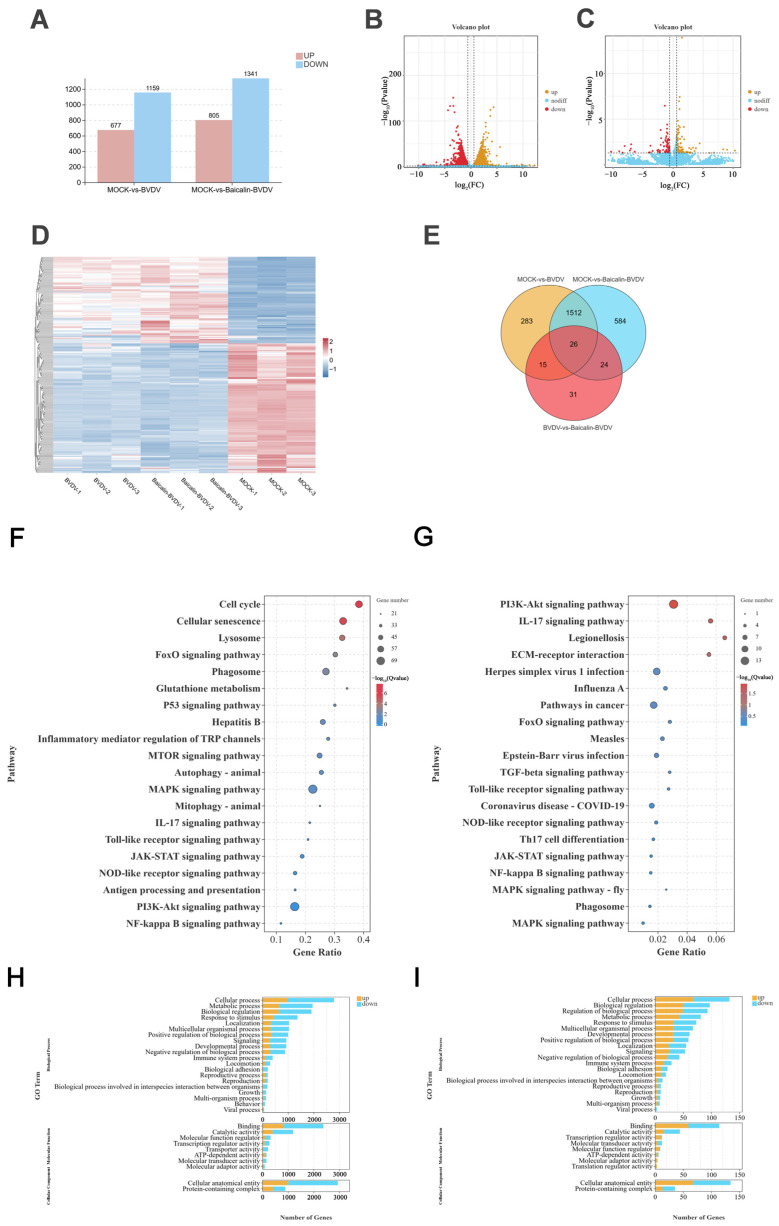
Transcriptomics analysis (*n* = 3). (**A**) Statistical graph of the result of differential analysis. (**B**,**C**) The figure shows the gene differences between the mock group and the BVDV group and between the BVDV group and the BA + BVDV group. Each point in the figure represents a gene, with red indicating upregulated genes, yellow indicating downregulated genes, and blue indicating genes with no difference in expression. (**D**) *DEGs* clustering heatmap. (**E**) Venn diagram: conduct intersection analysis of differential genes. (**F**,**G**) Significance bubble plots are used to display significantly enriched pathways among different genes in each comparison group. (**H**,**I**) Map differentially expressed genes to GO terms in the MF, CC, and BP categories of the GO database, and analyze the differentially expressed genes between the Mock group and the BVDV group and the BA + BVDV group.

Based on the prior finding that BA inhibits BVDV during the replication phase of its replication cycle, we subsequently performed cellular transcriptome sequencing on the control group, BA group, and BA+BVDV group to identify BA’s antiviral mechanism. Compared to the control group, BVDV-infected cells exhibited 1836 differentially expressed genes (*DEGs*) (677 upregulated, 1159 downregulated); BA-treated cells exhibited 2146 *DEGs* (805 upregulated, 1341 downregulated) relative to controls (Figure 4A–C). Differential gene screening employed thresholds of FDR < 0.05 and |log_2_FC| > 1. Clustering heatmap analysis revealed significant differences in transcriptomic profiles among experimental groups (Figure 4D,E), confirming BA-induced transcription during viral infection and establishing a foundation for subsequent mechanistic studies. KEGG and GO analyses characterized the differentially expressed genes. GO enrichment analysis revealed that downregulated *DEGs* in BVDV-infected cells were concentrated in immune regulation and apoptosis pathways, while upregulated *DEGs* were enriched in cellular metabolism and structural components (Figure 4H,I). This indicates that BVDV employs immune evasion strategies during replication in the host. KEGG pathway analysis revealed significant enrichment of inflammatory signaling cascades, including NOD-like receptor, Toll-like receptor, JAK-STAT, MAPK, and NF-κB pathways (Figure 4F,G). Notably, the NOD-like receptor pathway emerged as a central regulator of virus-induced inflammatory responses. Biological process analysis further linked *DEGs* to immune modulation, ATP metabolism, and antioxidant activity. These findings warrant targeted experimental validation to elucidate BA’s antiviral mechanism at the molecular level.

### 3.5. BA-PEG-PLGA NPs Modulate BVDV-Induced Autophagy to Suppress Inflammatory Cytokine Expression

**Figure 5:**
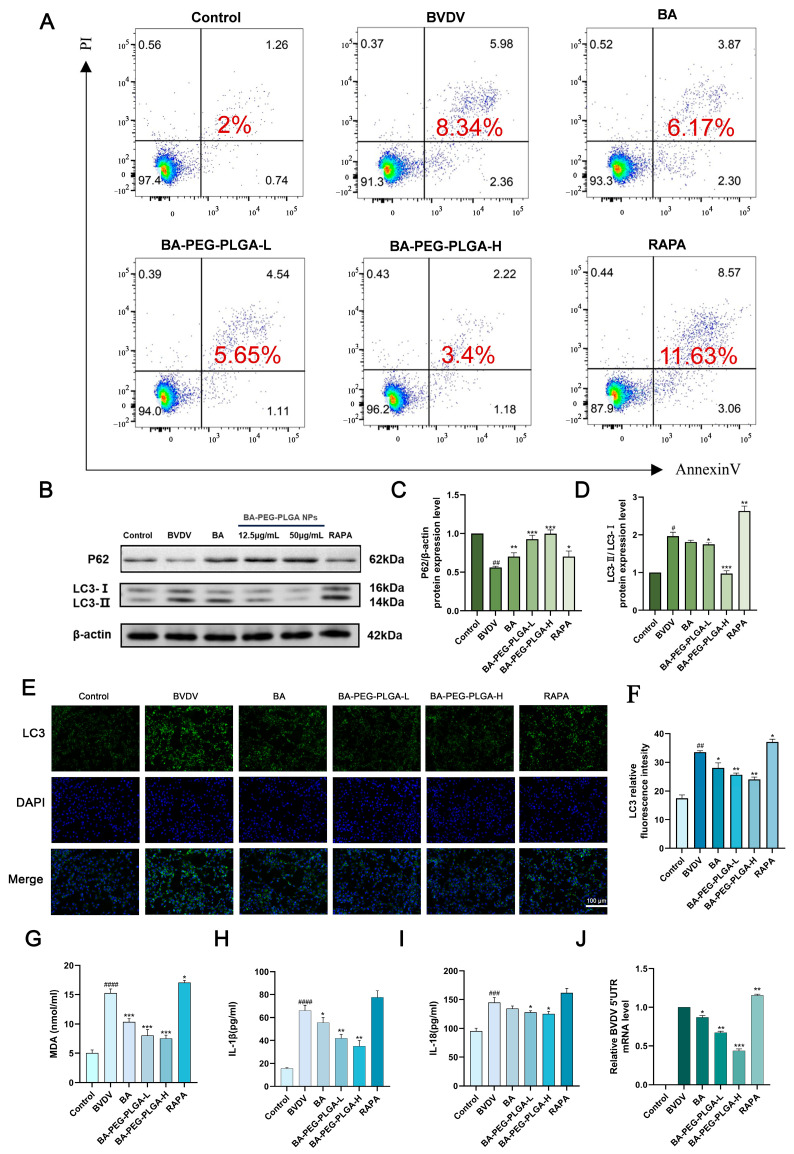
BA-PEG-PLGA NPs inhibit BVDV-induced autophagy to promote inflammatory cytokine expression. (**A**) The effects of BA, BA-PEG-PLGA NPs, and RAPA on MDBK apoptosis were detected by flow cytometry, The red numbers indicate the total apoptotic cells. (**B**) Protein expression of P62 and LC3-II/I was analyzed via Western blot. (**C**,**D**) Densitometric quantification of Western blot bands was performed on MDBK cell samples. (**E**) Autophagy was visualized by LC3 (green) immunofluorescence staining with DAPI (blue) nuclear counterstaining. Scale bar = 100 μm. (**F**) LC3 relative fluorescence intensity in MDBK cells treated (*n* = 3). (**G**–**I**) BA-PEG-PLGA nanoparticles suppressed IL-1β, IL-18, and MDA levels in MDBK cells. (**J**) BVDV mRNA expression levels in MDBK cells with BVDV infection and treated with BA, BA-PEG-PLGA, or RAPA. (*n* = 3, # *p* < 0.05, ## *p* < 0.01, ### *p* < 0.001, #### *p* < 0.001 vs. control; * *p* < 0.05, ** *p* < 0.01, *** *p* < 0.001 vs. BVDV). Western blot originals can be found in Figure S2.

Previous research has demonstrated that BVDV can induce autophagy to evade immune detection and promote viral replication. We investigated whether BA and its nanoparticles exert anti-BVDV activity by inhibiting autophagy. BA, BA-PEG-PLGA-L, BA-PEG-PLGA-H, and RAPA treatments were administered following BVDV infection. We measured the expression levels of autophagy-related marker proteins (including LC3I, LC3II, and P62, which serves as an autophagy flux marker) in cells infected with BVDV. When cells are stimulated by the external environment, apoptosis is triggered and may produce inflammatory factors. Flow cytometry was performed to assess cell viability and apoptosis rate in virus-infected cells following drug treatment, using annexin V/PI double staining. The results (Figure 5A) showed that the total apoptosis rate was 2% in the control group, 8.34% in the BVDV group, and 11.63% with the addition of RAPA (autophagy inducer), which exacerbated the BVDV-induced apoptosis. Baicalin and BA-PEG-PLGA can enhance cell viability and promote orderly cell proliferation. Results showed that compared with the control group, the BVDV-infected group exhibited increased LC3II/I protein expression and decreased SQSTM1/p62 expression, whereas the BA-PEG-PLGA-H group significantly reduced LC3II/I protein expression and increased SQSTM1/p62 protein expression levels, indicating that the drug group suppressed virus-induced complete autophagy flux (Figure 5B–D). This indicates that BA, which regulates BVDV-induced autophagy, may prevent autophagy degradation in MDBK cells. Subsequently, immunofluorescence analysis revealed that BA and BA-PEG-PLGA treatment groups significantly reduced the fluorescence intensity of LC3, an autophagy-associated protein induced by BVDV (Figure 5E). Malondialdehyde (MDA), a common lipid peroxide, serves as an effective indicator for detecting cellular oxidative stress. Viral infection significantly elevated cellular MDA levels, and ELISA detection revealed markedly increased secretion of IL-1β and IL-18 in cell culture supernatants. Pharmacological intervention effectively alleviated the inflammatory response (Figure 5G–I). These findings indicate that BA and BA-PEG-PLGA can modulate oxidative stress and inflammatory responses induced by bovine viral diarrhea virus. Among these, BA-PEG-PLGA nanoparticles demonstrated superior therapeutic efficacy compared to BA. This advantage stems from the nanoparticle formulation’s ability to delay drug release, span multiple viral replication cycles, reduce drug loss, and enhance therapeutic outcomes.

### 3.6. BA-PEG-PLGA Inhibits Pyroptosis in Virus-Infected Cells by Suppressing NLRP3/Caspase-1 Inflammasome Activation

**Figure 6:**
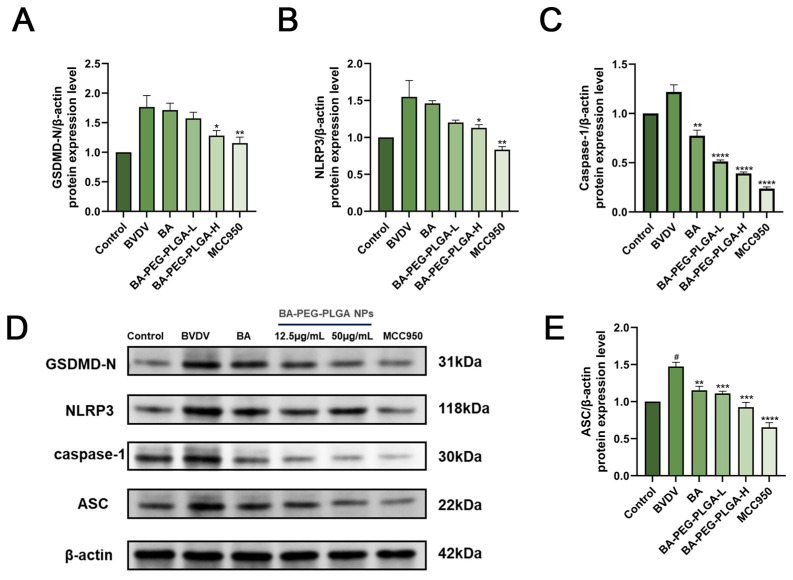

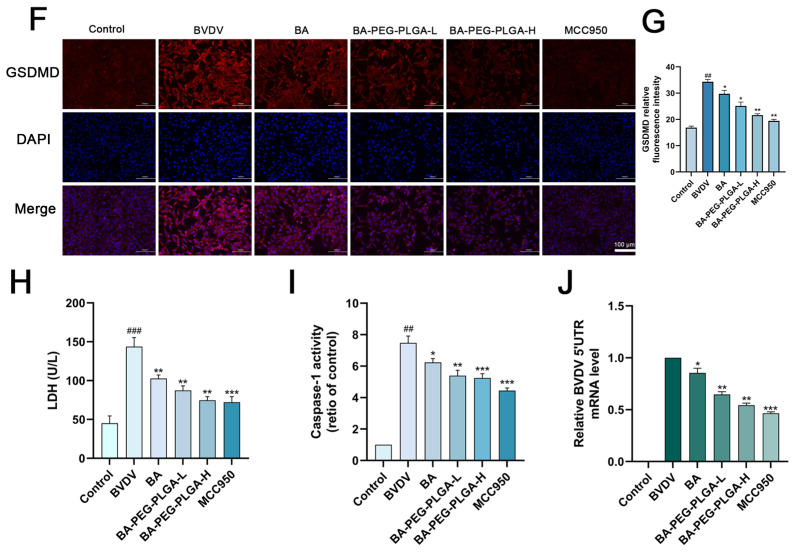
Baicalin inhibits pyroptosis in virus-infected cells by suppressing NLRP3/Caspase-1 inflammasome activation. (**A**–**E**) Western blot analysis of pyroptosis markers: (**A**–**C**) Quantitative protein levels of GSDMD-N, NLRP3, and Caspase-1; (**E**) ASC protein expression. (**F**) Pyroptosis visualization via GSDMD (red) immunofluorescence staining (DAPI: blue nuclei; scale bar = 100 μm). (**G**) GSDMD relative fluorescence intensity in MDBK cells treated (*n* = 3). (**H**) LDH activity in culture supernatants and (**I**) Caspase-1 activation levels post-BVDV infection. BA-PEG-PLGA NPs and NLRP3 inhibitor MCC950 showed comparable efficacy in reducing inflammatory markers. (**J**) BVDV mRNA expression levels in MDBK cells with BVDV infection and treated with BA, BA-PEG-PLGA, or MCC950. Data represent three independent experiments. (*n* = 3, # *p* < 0.05, ## *p* < 0.01, ### *p* < 0.001 indicates comparison with control; * *p* < 0.05, ** *p* < 0.01, *** *p* < 0.001, **** *p* < 0.0001 indicates comparison with BVDV). Western blot originals can be found in Figure S3.

NOD-like receptor protein 3 (NLRP3) inflammasome mediates the processing and secretion of interleukin-1β (IL-1β) [38,39]. BA, BA-PEG-PLGA-L, BA-PEG-PLGA-H, and MCC950 treatments were administered following BVDV infection. The results indicate that BVDV infection activates IL-18 and IL-1β secretion (*p* < 0.01). This further confirmed that BVDV infection could trigger a strong inflammatory response in the body. To investigate the effects of BA and its nanoparticles on the NLRP3 pathway in BVDV-infected cells, the NLRP3 inhibitor MCC950 was introduced in subsequent experiments. Western blot analysis (Figure 6A–E) revealed markedly elevated pyroptosis markers (GSDMD-N, NLRP3, Caspase-1, ASC) in infected cells, a phenomenon reversed by BA-PEG-PLGA treatment (*p* < 0.01). This effect was consistent with the action of NLRP3 inhibitor MCC950, indicating dual regulation of viral inflammation and pyroptosis cell death. The immunofluorescence experiments (Figure 6F,G) provided further evidence for the above-mentioned mechanism. The experimental results showed that the fluorescence intensity of GSDMD in BVDV-infected cells was enhanced, which was consistent with the increase in GSDMD-N in Western blot analysis. After treatment with BA-PEG-PLGA, the enhancement effect of GSDMD fluorescence intensity was inhibited, which further proved that BA-PEG-PLGA had an inhibitory effect on pyroptosis induced by viral infection.

In addition to the effects on GSDMD and pyroptosis markers, BA-PEG-PLGA also inhibited the activation of Caspase-1 and the release of lactate dehydrogenase (LDH) (*p* < 0.01) (Figure 6H–J). Caspase-1 is a key downstream effector molecule after the activation of the NLRP3 inflammasome, and its activation is closely related to the secretion of IL-1β and IL-18 and the occurrence of pyroptosis. LDH is an enzyme inside cells and will be released outside the cells when the cells are damaged. Therefore, the release amount of LDH is an important indicator to measure the degree of cell damage. The inhibitory effect of BA-PEG-PLGA on these two indicators further proved that it could inhibit the inflammatory response and cell damage induced by viral infection and exert its antiviral and anti-inflammatory effects at multiple levels (*p* < 0.01).

### 3.7. BA-PEG-PLGA Inhibits the Regulation of the ROS/NLRP3/Pyroptosis Signaling Axis in Cells During Viral Infection

**Figure 7:**
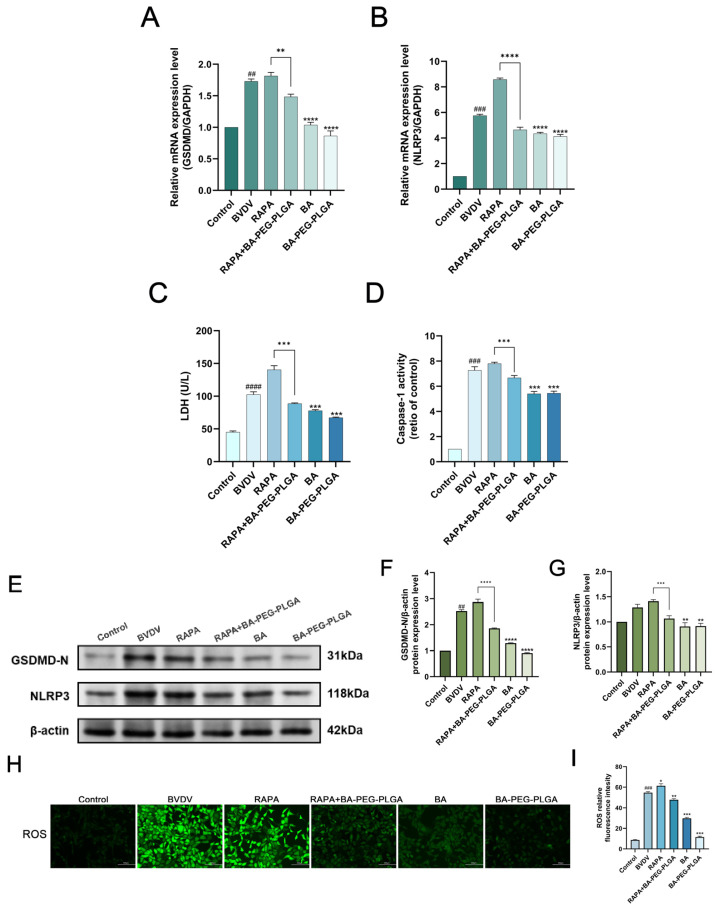
Scutellarin inhibits the regulation of the ROS/NLRP3/pyroptosis signaling axis in cells during viral infection. BA-PEG-PLGA modulates pyroptosis markers in BVDV-infected cells. (**A**,**B**) RT-qPCR analysis of GSDMD-N and NLRP3 mRNA levels. (**C**) LDH activity in culture supernatants. (**D**) Caspase-1 activation levels post-BVDV infection across treatment groups (BA, BA-PEG-PLGA NPs, RAPA autophagy inducer). (**E**–**G**) Western blot quantification of GSDMD-N and NLRP3 protein expression. (**H**) ROS detection in MDBK cells (scale bar = 100 μm). (**I**) GSDMD relative fluorescence intensity in MDBK cells treated (*n* = 3). Data represent three independent experiments (*n* = 3). Statistical significance: (*n* = 3, ## *p* < 0.01, ### *p* < 0.001, #### *p* < 0.001 vs. control; * *p* < 0.05, ** *p* < 0.01, *** *p* < 0.001, **** *p* < 0.0001 vs. RAPA). Western blot originals can be found in Figure S4.

BVDV induces intense inflammatory responses, further exacerbating the cytopathic effects of BVDV infection [40,41]. Numerous studies have shown that autophagy and inflammation are closely related [42]. With the aim of ascertaining whether autophagy triggered by BVDV is capable of affecting inflammation-mediated pyroptosis through the NLRP3 inflammasome. RAPA, RAPA+BA-PEG-PLGA, BA, and BA-PEG-PLGA treatments were administered following BVDV infection. NLRP3 and GSDMD expression were evaluated at mRNA and protein levels in BVDV-infected cells. BA-PEG-PLGA treatment reversed RAPA-induced effects, suppressing NLRP3 inflammasome activation alongside GSDMD-N transcription and protein expression (*p* < 0.01) (Figure 7A,B,E–G). During bovine viral diarrhea virus (BVDV) infection, intracellular ROS levels increased significantly, indicating that viral infection triggered an oxidative stress response in cells. BA-PEG-PLGA NPs reduced ROS levels. Additionally, the nanoparticles suppressed RAPA-enhanced caspase-1 activation and lactate dehydrogenase (LDH) release (*p* < 0.01) (Figure 7C,D,H). This indicates that the reduction in intracellular ROS mediated by BA-PEG-PLGA depends on the autophagy pathway. Considering all results comprehensively, BA-PEG-PLGA can inhibit autophagy, reduce ROS production, alleviate oxidative stress, and help regulate ROS/NLRP3 inflammasome/pyroptosis-related signaling pathways in cells during viral infection.

### 3.8. BA-PEG-PLGA Modulation of NLRP3 Inflammasome Reduces Viral Load and Alleviates Virus-Induced Tissue Damage in BVDV-Infected Mice

**Figure 8:**
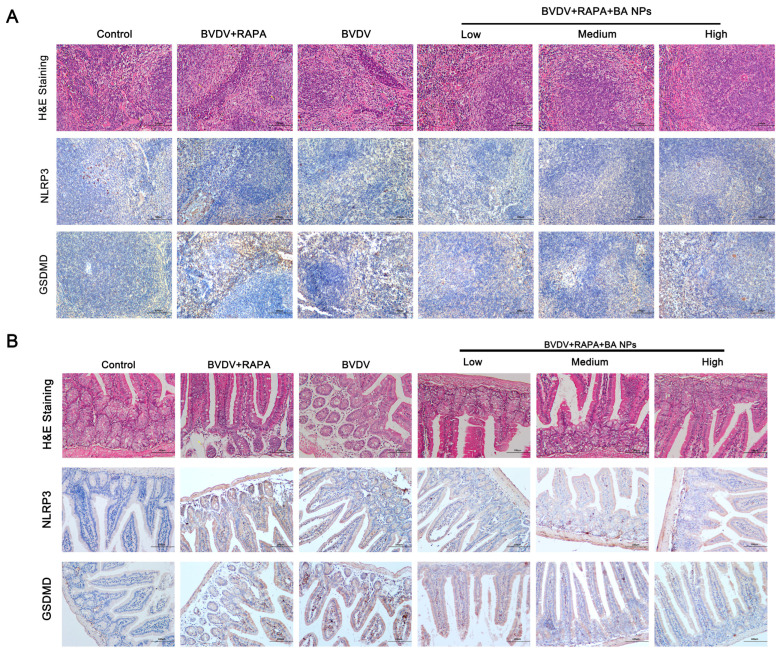
(**A**) Histological staining results of mouse spleen tissue, including hematoxylin and eosin (HE) staining, as well as immunohistochemical results for NLRP3 and GSDMD. (**B**) Histological staining results of mouse duodenal tissue, including hematoxylin-eosin (HE) staining, as well as immunohistochemical results for NLRP3 and GSDMD.

To better understand the mechanism of BA-PEG-PLGA’s protective effect in vivo, BALB/c mice (4–6 weeks old) were intraperitoneally injected with 200 μL viral suspension containing 10^5^ TCID_50_ BVDV and treated with low (5 mg/mL), medium (10 mg/mL), and high doses (20 mg/mL) of the drug. In the BVDV+RAPA group, the red arrow indicates compression marks where red pulp is squeezed by expanded white pulp (Figure 8A). Yellow arrows indicate submucosal edema in duodenal specimens, while red arrows indicate chronic inflammatory cell infiltration within the mucosal stroma (Figure 8B). BA-PEG-PLGA reduced these pathologies in a dose-dependent manner, with high doses significantly improving spleen and duodenal conditions and reducing inflammation.

**Figure 9:**
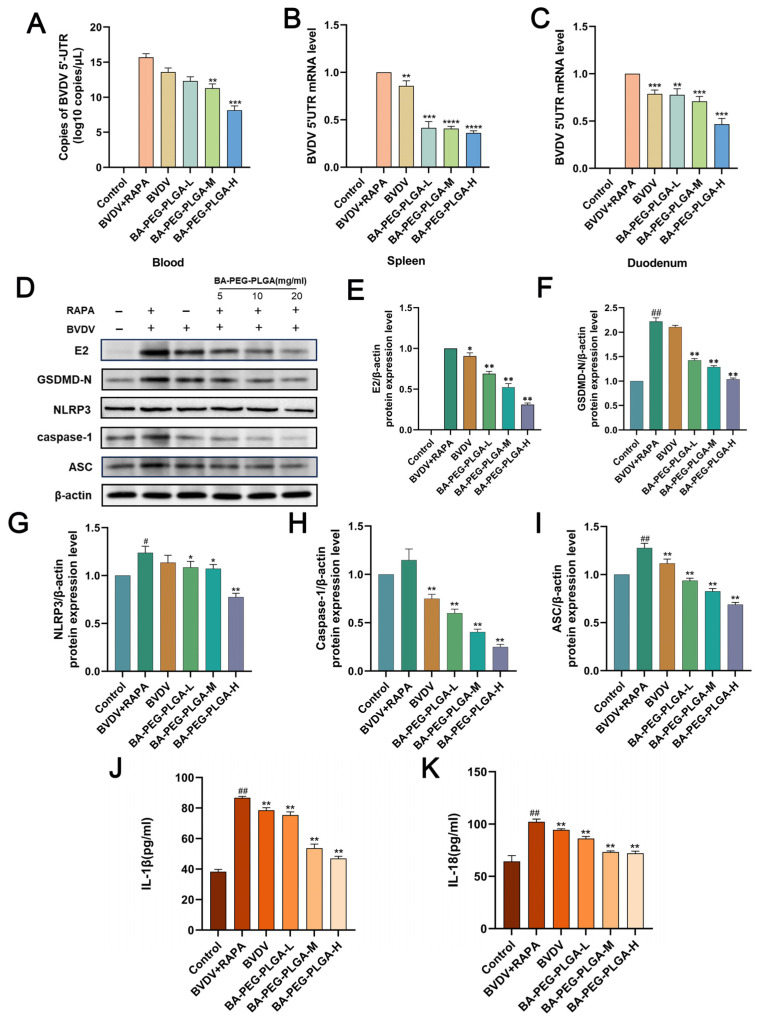
(**A**–**C**) Quantitative Fluorescent PCR Detection of Viral Load in Tissues and Blood from Six Groups of Mice Following Challenge. (**D**) Western blotting results of mouse spleen tissue. (**E**–**I**) Western blot density analysis of mouse spleen tissue. (**J**,**K**) In the blood of BALB/c mice, BA-PEG-PLGA lowers the amounts of inflammatory cytokines IL-1β and IL-18. Data represent three independent experiments (*n* = 3). Statistical significance: (*n* = 3, # *p* < 0.01, ## *p* < 0.01 indicates comparison with control; * *p* < 0.05, ** *p* < 0.01, *** *p* < 0.001, **** *p* < 0.0001 indicates comparison with BVDV+RAPA). Western blot originals can be found in Figure S5.

On day 7 of bovine viral diarrhea virus (BVDV) infection, tissue samples from the mouse spleen, duodenum, and blood were collected. Viral load was detected via RT-qPCR and Western blot analysis (Figure 9A–C). Results showed detectable BVDV in all intraperitoneally inoculated mice. Compared to the BVDV+RAPA group, BA-PEG-PLGA significantly suppressed rapamycin-promoted BVDV 5′UTR mRNA expression in the duodenum, spleen, and blood in a dose-dependent manner, while also significantly inhibiting viral E2 protein expression (Figure 9E). This indicates that BA-PEG-PLGA effectively suppresses autophagy-promoted BVDV replication. Additionally, compared to the control group, the BA-PEG-PLGA group significantly suppressed the increase in serum IL-1β and IL-18 levels induced by rapamycin (*p* < 0.01) (Figure 9J,K). The rapamycin group significantly upregulated NLRP3 and its downstream effector molecules (including ASC, Caspase-1, and GSDMD) at the protein level, whereas the BA-PEG-PLGA group reversed the effects observed in the rapamycin-virus co-treatment group. This suggests that baicalin nanoparticles may reduce viral load in BVDV-infected mice by inhibiting autophagy, NLRP3 inflammasome assembly, and GSDMD expression, thereby alleviating virus-induced inflammatory responses and mitigating pathological tissue damage in the spleen and duodenum, potentially yielding positive therapeutic effects against BVDV.

## 4. Discussion

Bovine viral diarrhea virus (BVDV), a member of the Pestivirus genus within the Flaviviridae family, imposes substantial economic burdens on global cattle industries through immunosuppression, reproductive failure, and mucosal disease [2,3,4]. While baicalin (BA) exhibits broad-spectrum antiviral activity, its clinical translation against BVDV has been hindered by poor aqueous solubility (~67 μg/mL) and extensive metabolic inactivation by gut microbiota, resulting in low systemic bioavailability [20,21]. Nanoparticles can be used as drug carriers, and nanoparticles are easily absorbed by cells, increasing drug accumulation and retention time, thus further improving the effectiveness of antiviral therapy. Therefore, we engineered BA-loaded PEG-PLGA nanoparticles (BA-PEG-PLGA NPs) with uniform spherical morphology (130 nm) and sustained release kinetics (94.2% cumulative release within 48 h). Encapsulating BA within the PEG–PLGA matrix enhances its solubility and cumulative release efficiency. Critically, the application of BA-PEG-PLGA NPs for combating pestivirus infection has not been previously reported.

Inducing autophagy to promote viral replication has been recognized as a mechanism utilized by flaviviruses to evade host immune responses and establish persistent infections. Classical swine fever virus (CSFV) specifically relies on the autophagy pathway during replication and maturation, utilizing autophagic membranes as scaffolds for viral assembly within host cells [43]. This virus disrupts normal autophagy function, leading to excessive activation of the inflammatory response. Previous studies have demonstrated that BVDV replication in cells is associated with autophagosomes, as confirmed by immunolocalization of the BVDV autophagy marker LC3 [44]. BVDV infection drives cells to complete the full autophagy flux [45]. This study confirmed, through in vitro LC3 immunofluorescence and Western blotting experiments, that an increased LC3II/LC3I ratio (particularly accompanied by reduced p62 accumulation) indicates the induction of a complete autophagic flux within infected cells during viral infection. This induced autophagy enhances cellular inflammatory factors, NLRP3 inflammasome activation, and pyroptosis processes. In vivo experiments yielded consistent results, demonstrating that BA-PEG-PLGA significantly mitigated rapamycin (RAPA)-induced effects while inhibiting viral replication both in vitro and in vivo. Notably, the functional consequence of BVDV-induced autophagy, its role in driving inflammatory pathology, remained uncharacterized prior to our work. Our data reveal that BVDV-triggered autophagy does not merely support viral replication but actively exacerbates host damage by promoting reactive oxygen species (ROS) overproduction and subsequent inflammatory cascade activation.

Autophagy regulates inflammatory signaling through multiple pathways, thereby driving the progression of inflammatory responses [46]. The viral replication process triggers a series of cellular alterations that activate inflammasomes, including viral protein aggregation, endosomal rupture, reactive oxygen species (ROS) production, ATP release, ion current imbalance, and direct or indirect interactions with viral proteins during replication [47,48]. Excessive accumulation of reactive oxygen species (ROS) causes tissue damage by disrupting lipid and protein structures while inducing apoptosis. Taking Newcastle disease virus infection as an example, the infection process promotes excessive intracellular calcium ion accumulation and generates large amounts of ROS [49], with oxidative stress causing systemic damage [50]. The NLRP3 inflammasome is a key protein in immune regulation. Upon activation by pathogens or cellular stress signals, it triggers pyroptosis, a lytic form of programmed cell death through caspase-1-mediated cleavage of gasdermin D (GSDMD) and maturation of pro-inflammatory cytokines such as IL-1β and IL-18 [51,52]. Differential gene analysis in transcriptomics in BVDV-infected MDBK cells primarily clusters within pyroptosis, NOD-like receptor, and RIG-I-like receptor signaling pathways [53,54]. The NLRP3/Caspase-1/GSDMD-N axis is crucial in regulating pyroptosis, making NLRP3 inflammasome inhibition a potential therapeutic strategy for inflammatory diseases.

Building on this foundation, we uncovered a previously unreported pathogenic axis in BVDV infection: the autophagy–ROS–NLRP3/pyroptosis cascade. We demonstrated that baicalin and its nanoparticles significantly inhibited the replication of BVDV. They also reduced intracellular inflammatory factors and attenuated virus-induced inflammatory damage to MDBK cells. Moreover, they inhibited the accumulation of intracellular ROS amplified by BVDV-induced autophagic flux, thus suppressing the NLRP3 inflammasome. This led to the caspase-1-dependent cleavage of gasdermin D (GSDMD) and the maturation of IL-1β/IL-18, which we proved to be hallmarks of the inhibition of pyroptotic cell death. In the experiment, rapamycin (RAPA) was used as an induction control. Autophagy exacerbated the activation of NLRP3 and inflammatory damage, while the inhibition of NLRP3 with MCC950 attenuated these effects, further confirming the therapeutic effects of baicalin and its nanoparticles.

Both free BA and BA-PEG-PLGA NPs effectively suppressed BVDV replication and mitigated inflammatory injury; however, the nanoformulation exhibited superior efficacy attributable to enhanced bioavailability. Mechanistically, BA and its nanoformulation coordinately targeted the identified pathogenic axis by: (i) restoring autophagy homeostasis (preventing excessive flux without complete blockade), (ii) scavenging ROS to interrupt oxidative stress signaling, and (iii) inhibiting NLRP3 inflammasome assembly and downstream GSDMD cleavage, with effects comparable to the specific NLRP3 inhibitor MCC950. These findings position BA not merely as a direct antiviral agent but as a host-targeted therapeutic that modulates virus–host interactions at the autophagy–inflammation interface.

In conclusion, developing effective anti-BVDV strategies is of great significance for the sustainable development of the cattle industry. Our research findings contribute to a better understanding of the complex interactions among the virus, host cells, and the immune system. Our findings also lead to the discovery of new mechanisms related to autophagy and inflammation in the context of BVDV infection. These findings elucidate the molecular mechanisms underlying the interaction between autophagy and inflammation during bovine viral diarrhea virus replication, providing future research directions for developing targeted antiviral drugs. While further studies are warranted to evaluate long-term safety and field efficacy, our findings provide a foundation for developing flavonoid-based interventions targeting autophagy–inflammasome cross-talk in viral inflammatory diseases.

## Figures and Tables

**Table 1:** MDBK cell RT-qPCR-related primer sequences.

Gene	Forward (5′-3′)	Reverse (5′-3′)
*GSDMD*	GCTGGTTATTGGCTCTGACTGG	ACGGATGTGGATGGCTGTCTG
*NLRP3*	TCACCAGGCTGCGTCTCATC	TCACAGAACTCACAGGGCTATCC
*GAPDH*	ACGGCACAGTCAAGGCAGAG	CACATACTCAGCACCAGCATCAC
*BVDV*	CATGCCCATAGTAGGAC	CCATGTGCCATGTACAG

## Data Availability

All relevant data are within the manuscript.

## Supplementary Materials

The following supporting information can be downloaded at https://www.mdpi.com/article/10.3390/biom16040502/s1. Figure S1. Original blots of BA-PEG-PLGA NPs suppressed the protein E2 in BVDV-infected cells in Figure 2. Figure S2. Original blot images of the expression of autophagy proteins P62 and LC3-II/I in MDBK cells infected with BVDV treated with BA-PEG-PLGA NPs in Figure 5. Figure S3. Original blots showing that BA-PEG-PLGA NPs suppressed pyroptosis markers (GSDMD-N, NLRP3, Caspase-1, ASC proteins) in BVDV-infected cells in Figure 6. Figure S4. Original blot images of the expression of GSDMD-N and NLRP3 in cells infected with BVDV treated with BA-PEG-PLGA NPs in Figure 7. Figure S5. Original blots of BA-PEG-PLGA NPs suppressed the NLRP3 inflammasome pathway (E2, GSDMD-N, NLRP3, Caspase-1, ASC proteins) in mouse spleen tissue in Figure 9.

## Abbreviations

The following abbreviations are used in this manuscript:

BVDV	Bovine viral diarrhea virus
BA	Baicalin
BA-PEG-PLGA	Baicalin-polyethylene glycol-polylactic acid-polyethylene glycol
MDBK	Madin–Darby Bovine Kidney cells
RAPA	Rapamycin
MCC950	CRID3sodium salt
NLRP3	NOD-like receptor family, pyrin domain-containing 3
qPCR	Quantitative Polymerase Chain Reaction
TCID_50_	50% Tissue Culture Infective Dose
CPE	Cytopathic Effect
ECL	Enhanced Chemiluminescence
LDH	Lactate dehydrogenase
FTIR	Fourier transform infrared
XRD	X-ray diffraction analysis
UV-vis	Ultraviolet–visible spectroscopy
