# Supplementary material for: PEG-PLGA Co-Loaded Baicalin Mitigates Bovine Viral Diarrhea Virus-Induced Oxidative Stress and Inflammatory Responses Through Modulation of Autophagy and Attenuation of the NLRP3/Pyroptosis Regulatory Axis
Source: Biomolecules. 2026 Mar 27;16(4):502. doi: 10.3390/biom16040502 (PMC13113166; doi:10.3390/biom16040502)
Supplement: Supplementary file 1 [file biomolecules-16-00502-s001.zip › biomolecules-4100053-supplementary.pdf]

Supplementary Material

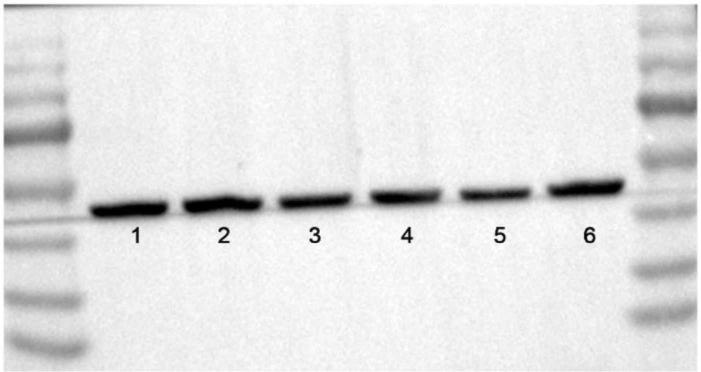

1. Control 2. BVDV 3. BA 4. BA-PEG-PLGA(12.5μg/mL) 5. BA-PEG-PLGA(25μg/mL) 6.BA-PEG-PLGA(50μg/mL)

β-actin

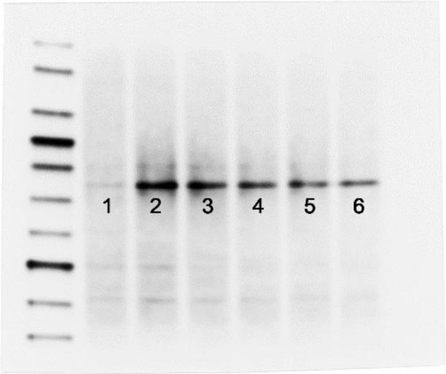

E2

Figure S1. Original blots of BA-PEG-PLGA NPs suppressed the protein E2 in BVDV-infected cells in Figure 2.

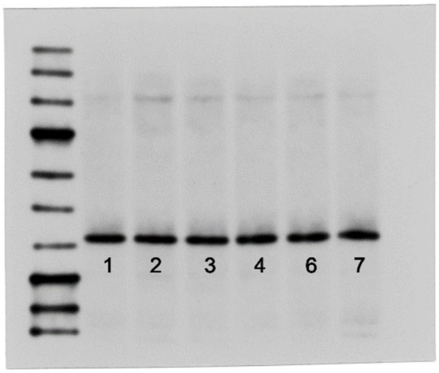

1.Control 2.BVDV 3. BA 4. BA-PEG-PLGA-l 5.BA-PEG-PLGA-H 6. RAPA

β-actin

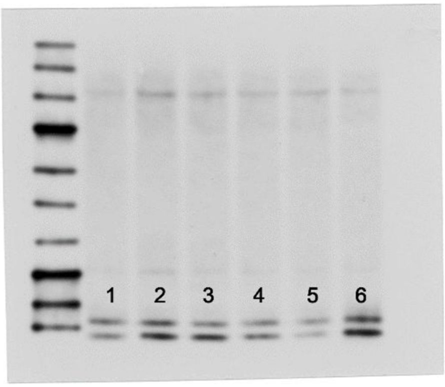

LC3

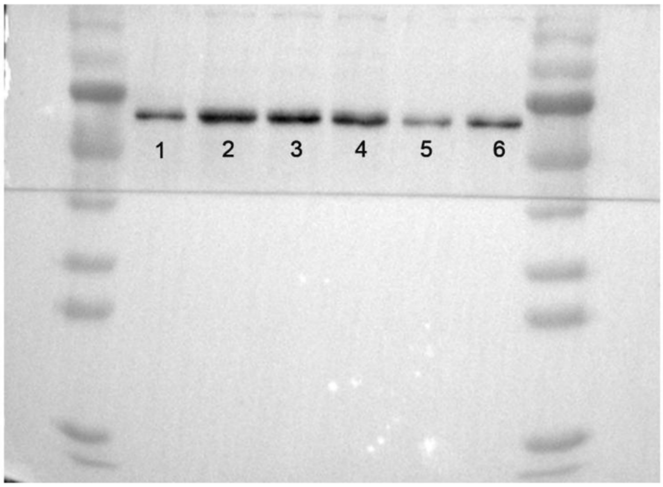

P62

Figure S2. Original blot images of the expression of autophagy proteins P62 and LC3-II/I in MDBK cells infected with BVDV treated with BA-PEG-PLGA NPs in Figure5.

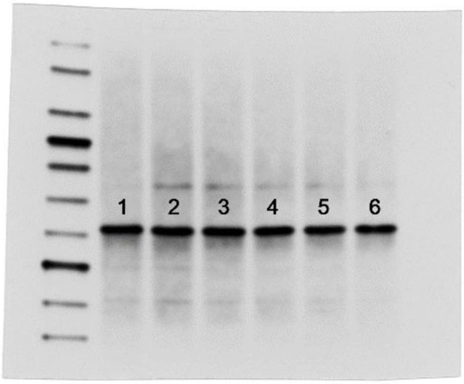

1. Control 2.BVDV 3. BA 4. BA-PEG-PLGA-L 5. BA-PEG-PLGA-H 6.MCC950

β-actin

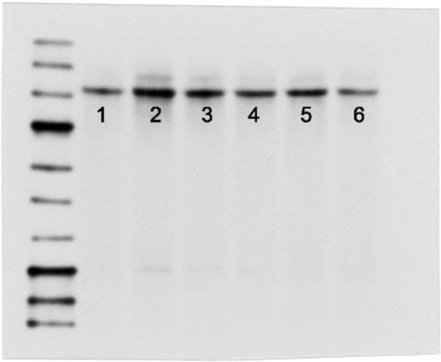

NLRP3

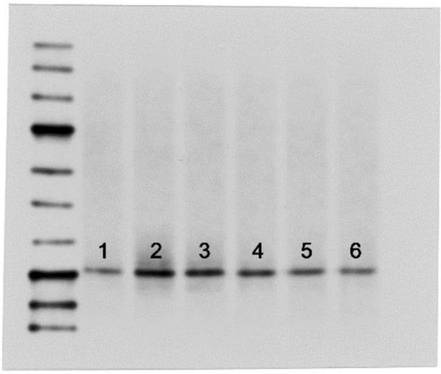

GSDMD-N

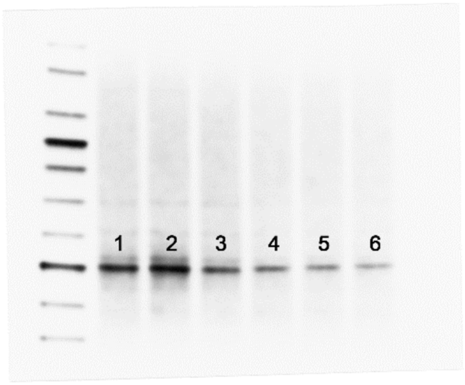

Caspase-1

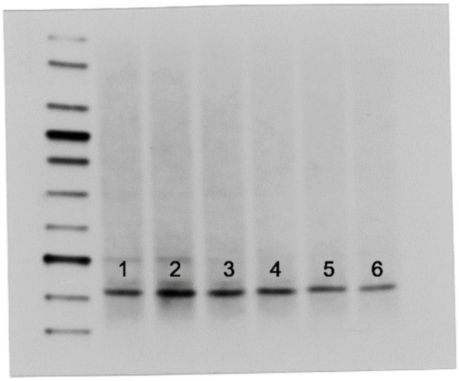

ASC

Figure S3. Original blots showing that BA-PEG-PLGA NPs suppressed pyroptosis markers (GSDMD-N, NLRP3, Caspase-1, ASC proteins) in BVDV-infected cells in Figure 6.

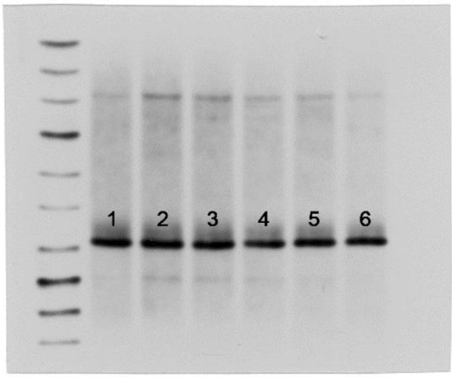

1. Control 2. BVDV 3.RAPA 4. RAPA+BA-PEG-PLGA 5. BA-PEG-PLGA 6.BA

β-actin

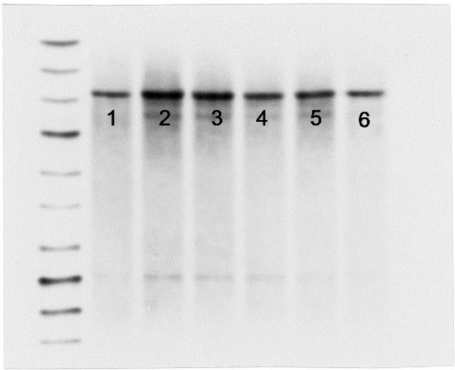

NLRP3

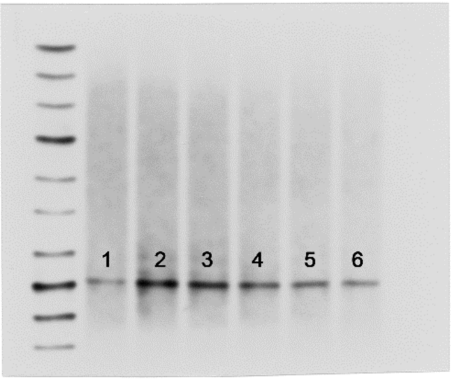

GSDMD-N

Figure S4. Original blot images of the expression of GSDMD-N and NLRP3 in cells infected with BVDV treated with BA-PEG-PLGA NPs in Figure 7.

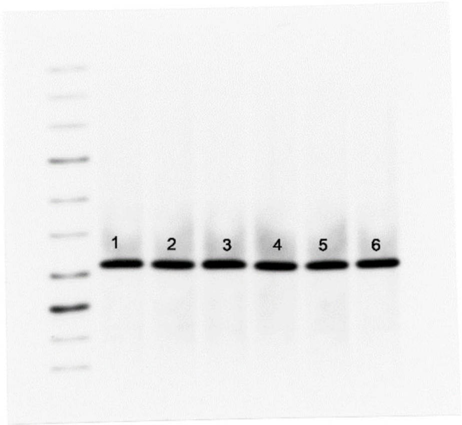

1. Contol 2. BVDV+RAPA 3. BVDV 4.BA-PEG-PLGA-L(5mg/ml) 5.BA-PEG-PLGA-M(10mg/ml) 6. BA-PEG-PLGA-L(20mg/ml)

β-actin

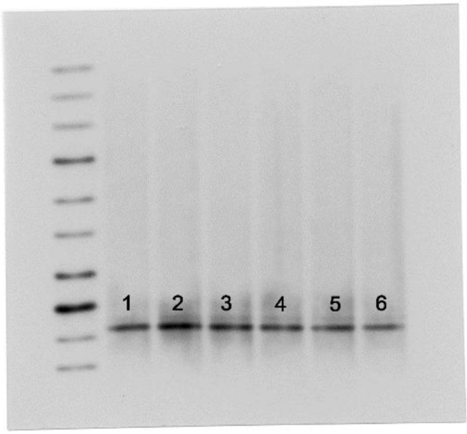

ASC

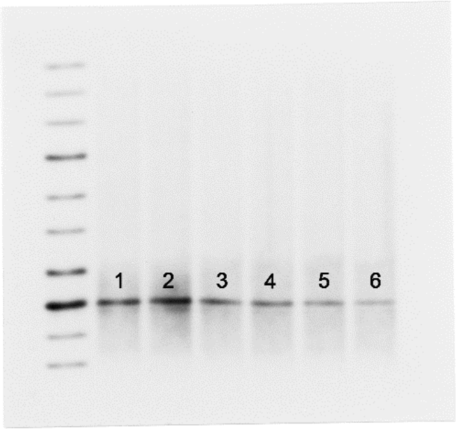

Caspase-1

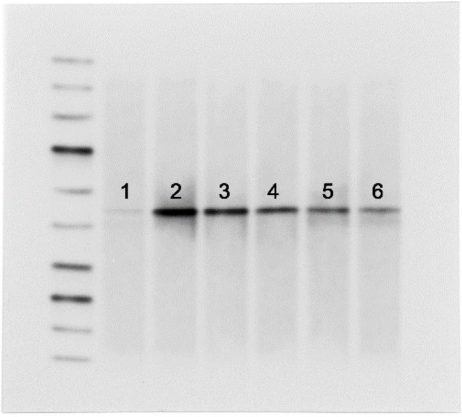

E2

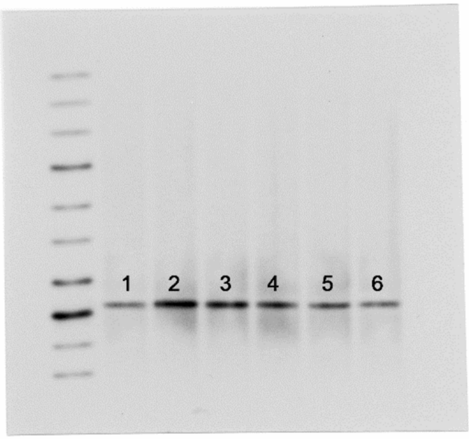

GSDMD-N

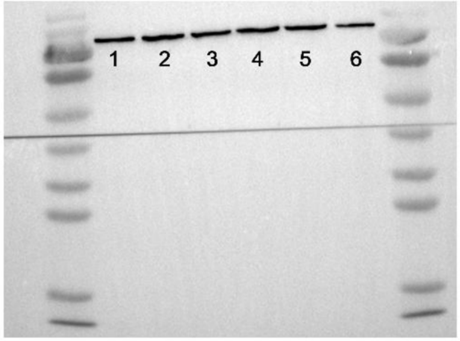

NLRP3

Figure S5. Original blots of BA-PEG-PLGA NPs suppressed the NLRP3 inflammasome pathway (E2, GSDMD-N, NLRP3, Caspase-1, ASC proteins) in mouse spleen tissue in Figure 9.
